# Spontaneous Coronary Artery Dissection in a Postpartum Female: Case Report and Simplified Algorithm for its Diagnosis and Management

**DOI:** 10.7759/cureus.4387

**Published:** 2019-04-04

**Authors:** Nikhil Agrawal, Arjun Khunger, Kinan Dalal, Usman Zahid, Timothy Petrie

**Affiliations:** 1 Internal Medicine, University at Buffalo, Buffalo, USA; 2 Oncology, Cleveland Clinic, Cleveland, USA; 3 Cardiology, University at Buffalo, Buffalo, USA

**Keywords:** spontaneous coronary artery dissection, postpartum, pregnancy, interventional cardiology, angiography, ekg, transthoracic echocardiography, pci, cabg, medical treatment

## Abstract

Spontaneous coronary artery dissection is a rare, life-threatening cause of acute myocardial infarction that must always be considered in the differential diagnosis, particularly in young, healthy women with a paucity of typical risk factors for heart disease. We present a case of a 39-year-old White woman, three months postpartum, presenting with severe epigastric chest pain radiating to her neck. Subsequent workup using coronary angiography revealed spontaneous dissection of the distal left anterior descending artery. The patient was successfully managed by conservative treatment using low dose aspirin, metoprolol, and captopril, highlighting that hemodynamically stable patients with lesions in distal branches of coronary arteries and single-vessel disease can be successfully managed in a conservative fashion, without the need for surgical or percutaneous revascularization.

## Introduction

Spontaneous coronary artery dissection (SCAD) is a dissection of the coronary artery that is not related to trauma, iatrogenic causes, or atherosclerosis [1]. It is a highly unusual cause of acute coronary syndrome with a reported prevalence of 0.07% to 1% in the general population [2-4]. SCAD is most often associated with acute myocardial infarction (AMI) in women during their third trimester of pregnancy or the early postpartum period. Coronary dissection is thought to develop from either an intimal tear or a spontaneous hemorrhage in the vessel wall, forming a hematoma underneath the arterial wall and compressing the true coronary lumen leading to AMI. Early recognition and treatment is critical, given the high mortality rate associated with SCAD. We present the case of a 39-year-old female who presented to the hospital with chest pain and was found to have SCAD of the left anterior descending (LAD) artery on coronary angiography.

## Case presentation

A 39-year-old White female presented to the hospital with a three-day history of epigastric pain. She described her pain as a burning pain that started at rest and was radiating to her neck. Her pain was associated with shortness of breath, and she denied any episodes of nausea, vomiting, cough, fevers, chills, or sweating. She recently had a twin pregnancy and gave birth at about 32 weeks of gestation via caesarian section, but lost both her children after birth. This event had been very emotionally taxing on her for the past three months.

Her past medical history was only remarkable for gestational diabetes during her last pregnancy. She typically smoked one pack per day of cigarettes for the last 20 years. Recently, she increased her smoking to two packs per day for the past few months. She denied any use of alcohol or recreational drugs. She also denied any other cardiac risk factors, significant family history of cardiac disease, sudden cardiac death, or connective tissue disease. She had no allergies and was not taking any medications.

On examination, she was afebrile with a temperature of 36.9°C, heart rate of 82 beats/min, a blood pressure of 165/110, respiratory rate of 18 breaths/minute, and oxygen saturation of 99% on room air. Her head and neck examination were unremarkable with no jugular venous distention. On auscultation, there was good air entry bilaterally and normal heart sounds with no murmurs, rubs, or gallops. There was no edema on examination of the lower extremities.

**Figure 1:**
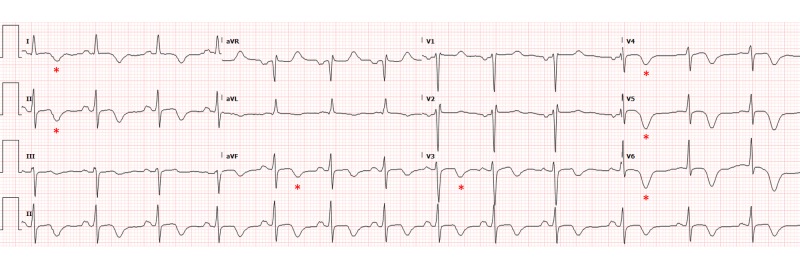
Electrocardiogram (EKG) on presentation showing sinus rhythm with sinus arrhythmia and T wave inversions concerning for anterior ischemia (asterisks).

An initial electrocardiogram (EKG) was obtained as can be seen in Figure 1.

**Figure 2:**
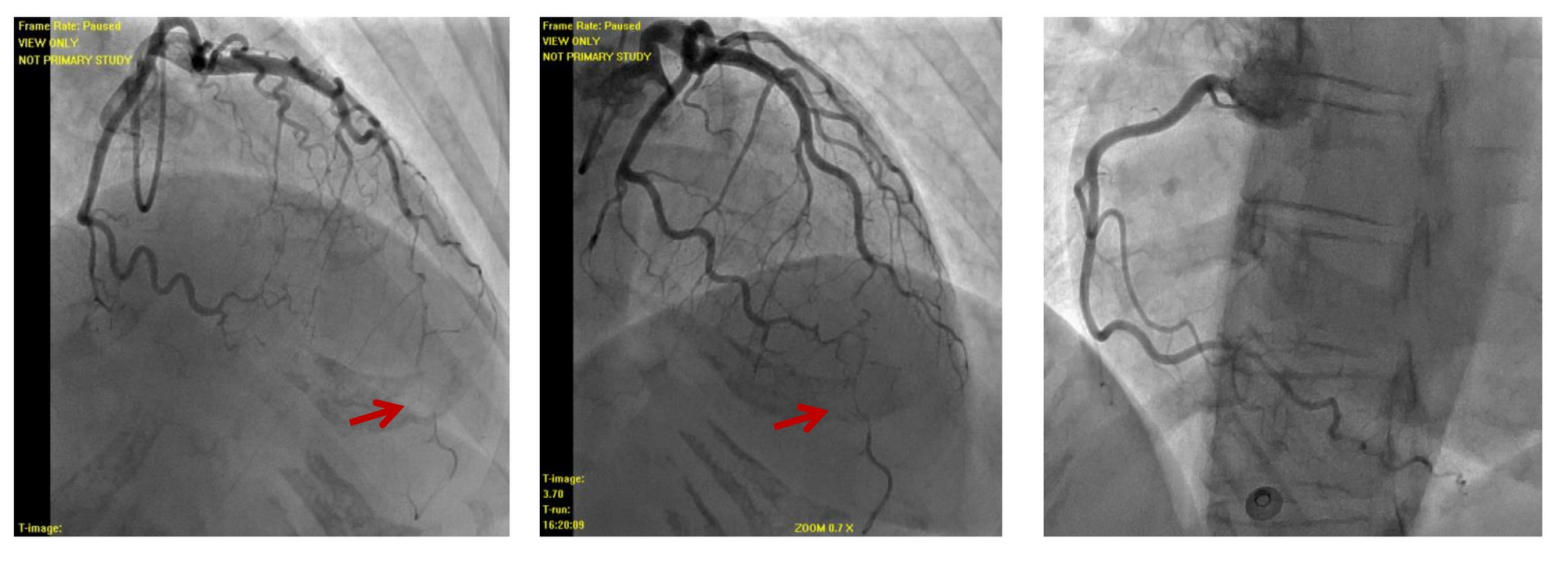
Angiography of the left coronary artery (left and middle image) and right coronary artery (right image). Left coronary angiography showing spontaneous coronary artery dissection of the distal left anterior descending artery with TIMI 2 flow (arrows). TIMI - Thrombolysis in Myocardial Infarction

**Figure 3:**
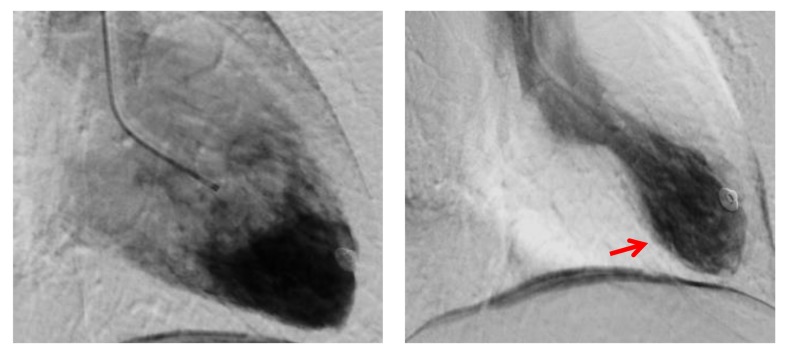
Angiography revealed akinetic apical segments (arrow) with an estimated left ventricular ejection fraction of 45%.

Her EKG showed sinus rhythm with sinus arrhythmia and anterolateral T wave inversions concerning for anterior ischemia. Cardiac biomarkers included a creatine kinase level of 261 U/L (normal range, 33-211 U/L), a creatine kinase-MB fraction of 13 U/L (normal, <10 U/L), and a troponin I level of 5.22 ng/mL (normal, <0.01 ng/mL). She was transferred from an urgent care facility after aspirin and ticagrelor loading for percutaneous coronary intervention. Her peak troponin I was 5.4. Angiography revealed spontaneous coronary artery dissection (SCAD) of the distal left anterior descending (LAD) artery without any other atherosclerotic disease (Figures 2-3). The other coronary arteries were normal. Catheterization also revealed a mildly reduced left ventricular ejection fraction of 45% and akinetic apical segments of the left ventricle.

**Figure 4:**
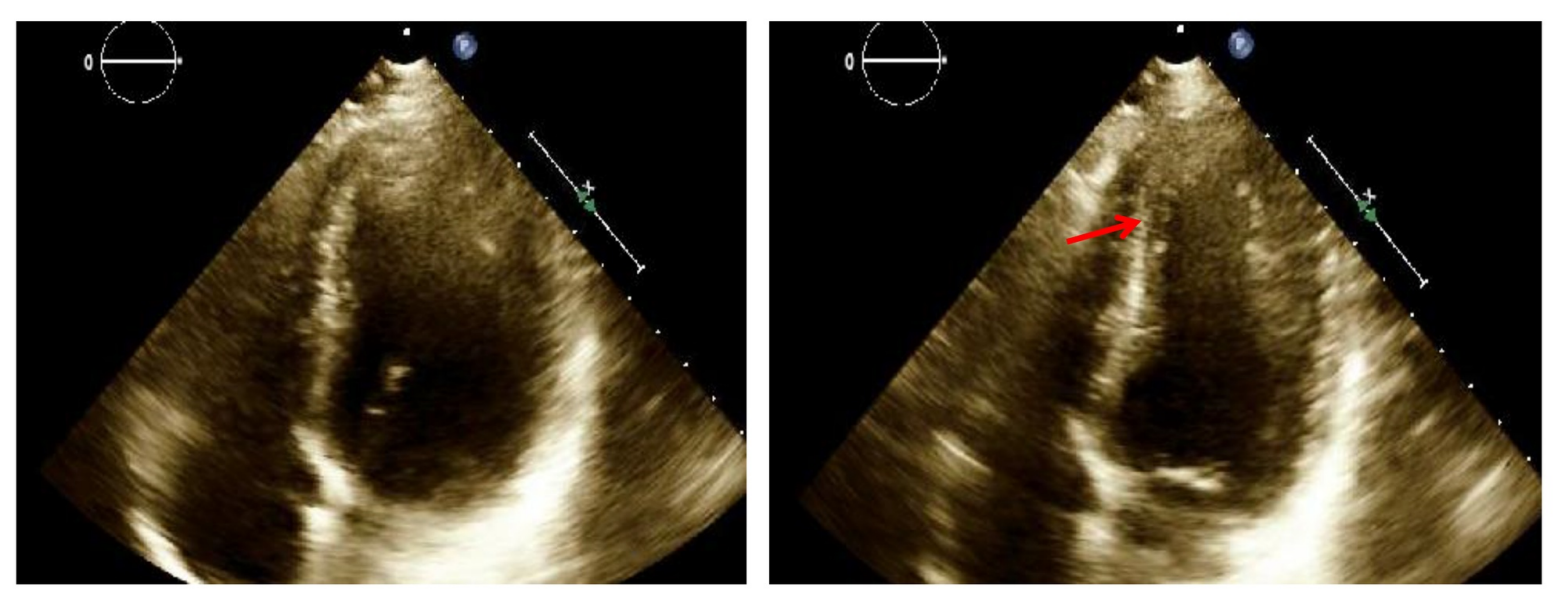
Transthoracic echocardiography on day 2 revealed regional wall motion abnormalities of the left ventricular apex with a low normal estimated left ventricular ejection fraction of 50%-55%. The right ventricle had normal systolic function and size. The left ventricle in four chamber view is shown in diastole (left) and systole (right) with an akinetic apex (arrow). No left ventricular thrombus was present.

She was started on medical management for SCAD, which included low dose aspirin, metoprolol, and captopril. Dual antiplatelet therapy and heparin were not given because of the risk for increased bleeding and no clear evidence of benefit. She was admitted to the cardiac critical care unit for observation and remained hemodynamically stable during her stay. Echocardiography done after one day confirmed wall motion abnormalities of the left ventricular apex with an estimated low normal left ventricular ejection fraction of 50%-55% (Figure 4). Laboratory workup for possible connective tissue disorders or vasculitis was done as underlying coronary vasculitis may increase the risk for spontaneous dissection and it was negative for rheumatoid factor, anti-CCP and ANA and only positive for atypical perinuclear anti-neutrophil cytoplasmic antibodies (P-ANCA). She was discharged two days after the presentation on medical therapy. The patient was doing well on the subsequent follow-up visit.

## Discussion

Spontaneous coronary artery dissection in the postpartum period is a rare but life-threatening event and several cases have been reported in the past [5-11]. The pathogenesis of pregnancy related-SCAD remains uncertain, and it is suggested that hormonal and hemodynamic changes during the peripartum period weaken the coronary artery walls, predisposing them to acute dissection. Women of advanced age are reported to have a higher risk of myocardial infarction (MI). Other risk factors for SCAD include systemic arteriopathies such as fibromuscular dysplasia, connective tissue disorders, extreme emotional stress, family history of SCAD, and, less frequently, coronary vasospasm and cocaine abuse [12,13].

Pregnancy-related SCAD is reported to occur up to three months after delivery in almost 80% of cases, at a median time of 13 days postpartum [14]. Dissection may be seen in any coronary artery; however, the LAD or left circumflex is involved in about 80% of cases [6]. The dissections may be multifocal in about 40% of the cases [6]. In this case, the combination of hormonal changes during pregnancy, advanced maternal age, history of severe emotional distress and smoking is the most likely etiology for this patient’s MI secondary to coronary artery dissection.

**Figure 5:**
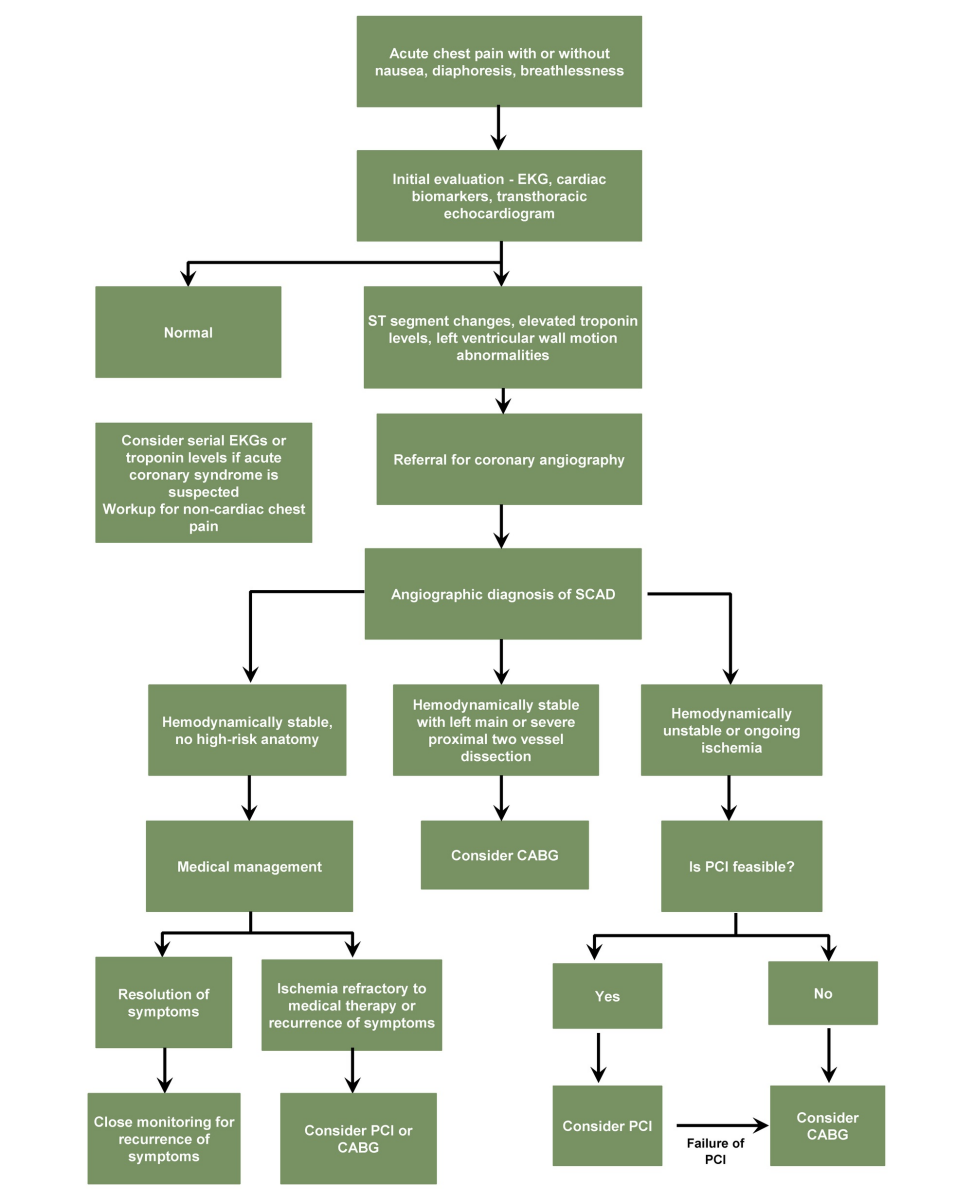
Algorithm for the diagnosis and management of spontaneous coronary artery dissection (SCAD) in a postpartum female CABG: Coronary Artery Bypass Graft; EKG: Electrocardiogram; PCI: Percutaneous Coronary Intervention; SCAD: Spontaneous Coronary Artery Dissection

Clinically, SCAD can present as stable or unstable angina, myocardial infarction, congestive heart failure, cardiac tamponade, cardiogenic shock, or sudden cardiac death [15,16]. As in the non-pregnant population, chest pain is the most common symptom at the initial presentation. Other common symptoms include nausea, diaphoresis, and breathlessness [12]. Initial evaluation includes an electrocardiogram, cardiac biomarkers, and a transthoracic echocardiogram. The diagnosis of SCAD is typically confirmed with the use of conventional coronary angiography. Figure 5 presents an algorithm for the diagnosis and management of SCAD in a postpartum female.

Management of SCAD should be individualized, based on the patient’s hemodynamic status, the site of dissection, and the extent of vessel involvement and may range from conservative medical therapy to percutaneous coronary intervention (PCI) or coronary artery bypass graft (CABG). For hemodynamically stable patients with lesions in distal branches of coronary arteries and single-vessel disease, medical treatment with antiplatelets (acetylsalicylic acid, clopidogrel), glycoprotein IIb/IIIa inhibitors, beta blockers, and nitroglycerin may be the best choice [17]. In patients with ongoing ischemia, unstable hemodynamics, or left main artery involvement, PCI or CABG should be considered [1]. PCI in SCAD is associated with complications such as extending the dissection and guidewires entering the false lumen [1]. In patients with unstable hemodynamics, multi-vessel involvement, failure of attempted PCI, or left main stem or proximal artery dissection, CABG is typically the treatment of choice [1]. Most of the studies discourage the use of fibrinolytic agents due to the risk of extension of the coronary dissection and worsening of any coronary spasm by compressing the vascular lumen [18]. Given the location of the dissection (distal LAD) and stable hemodynamic status of our patient, we chose to proceed with conservative therapy with low dose aspirin, metoprolol, and captopril. Further, recurrence of SCAD may be seen in 5.2% to 17% of patients, and they should be closely followed for any recurrent events or cardiac complications.

## Conclusions

Spontaneous coronary artery dissection (SCAD) should be strongly considered in the differential diagnosis for young women who present with signs and symptoms of acute coronary syndrome during pregnancy or the postpartum period. PCI has an increased risk of complications, and recurrence rates are high. Management requires a multidisciplinary approach and depends upon the clinical presentation, coronary anatomy, and hemodynamic stability of the patient.
